# Cortisol dysregulation in anxiety infertile women and the influence on IVF treatment outcome

**DOI:** 10.3389/fendo.2023.1107765

**Published:** 2023-06-13

**Authors:** Yujuan Chai, Qihang Li, Yang Wang, Ben Niu, Huijia Chen, Tingxuan Fan, Xiatong Ke, Heng Zou

**Affiliations:** ^1^ Department of Biomedical Engineering, School of Medicine, Shenzhen University, Shenzhen, Guangdong, China; ^2^ Department of Management, Shenzhen University, Shenzhen, Guangdong, China; ^3^ Reproductive Medicine Center, Department of Obstetrics and Gynecology, The Second Affiliated Hospital of Chongqing Medical University, Chongqing, China; ^4^ Greater Bay Area International Institute for Innovation, Shenzhen University, Shenzhen, Guangdong, China; ^5^ Research Department III, Shenzhen Health Development Research and Data Management Center, Shenzhen, Guangdong, China

**Keywords:** cortisol, anxiety, point-of-care testing, infertility, IVF outcome

## Abstract

**Introduction:**

Dysregulation of the stress-regulatory hormone cortisol is associated with anxiety, but its potential impact on infertile women and *in vitro* fertilization (IVF) treatment remains unclear. This prospective cross-sectional study aimed at evaluating the dysregulation of cortisol and its correlation to anxiety in infertile women. The influence of stress on IVF outcomes was also investigated.

**Methods:**

A point-of-care test was used for the measurement of morning serum cortisol in 110 infertile women and 112 age-matching healthy individuals. A Self-Rating Anxiety Scale (SAS) was used for the anxiety assessment of infertile women, and 109 of them underwent IVF treatment starting with the GnRH-antagonist protocol. If clinical pregnancy was not achieved, more IVF cycles were conducted with adjusted protocols until the patients got pregnant or gave up.

**Results:**

Higher morning serum cortisol level was identified for infertile patients, especially for the elder. Women with no anxiety showed significant differences in cortisol levels, monthly income, and BMI compared with those with severe anxiety. A strong correlation was found between the morning cortisol level and the SAS score. When the cutoff value is 22.25 μg/dL, cortisol concentration could predict the onset of anxiety with high accuracy (95.45%) among infertile women. After IVF treatments, women with high SAS scores (>50) or cortisol levels (>22.25 μg/dL) demonstrated a lower rate of pregnancy (8.0%-10.3%) and more IVF cycles, although the impact of anxiety was not affirmative.

**Conclusion:**

Hypersecretion of cortisol related to anxiety was prevalent among infertile women, but the influence of anxiety on multi-cycle IVF treatment was not affirmative due to the complicated treatment procedures. This study suggested that the assessment of psychological disorders and stress hormone dysregulation should not be overlooked. An anxiety questionnaire and rapid cortisol test might be included in the treatment protocol to provide better medical care.

## Introduction

Infertility is a global burden that affects 10%–15% of couples of reproductive age (1, 2). The impairment of reproductive function and stigmatization have led to a significantly higher level of stress and a prevalence of mood disorders (3–5). The overall prevalence of anxiety among infertile women was 36.17% (4), and a higher rate (37.2-42.2%) has been reported in Chinese patients who visited the reproductive medical center for help (6, 7). Anxiety will not only affect mental health but also impair reproduction function through complicated mechanisms such as dysregulation of hormones and metabolisms (8, 9). Therefore, the diagnosis of anxiety should not be overlooked for infertility treatment.

In addition to the classical scale tests for psychological assessment (10, 11), efforts have been made to identify biomarkers that can facilitate the evaluation of anxiety in infertile women (12, 13). The hypothalamus–pituitary–adrenal (HPA) axis hormones, especially cortisol, are major stress and threat regulators (14, 15). As one of the most abundant hormones in the human serum, cortisol regulates energy metabolism in response to adverse stimulations (16) and shares a common synthetic pathway with sex hormones such as testosterone and progesterone (17, 18). The complicated role of cortisol makes it a good target for neuroendocrine studies among women of reproductive age.

The impact of cortisol dysregulation on reproductive function and *in vitro* fertilization (IVF) has been well established. In spontaneous fertilization, cortisol could act directly on granulosa lutein cells to inhibit the support of steroidogenesis by luteinizing hormone (19). It also plays an important role in follicular development and safeguards oogenesis by promoting follicular cell survival (20). Increased levels of cortisol and prolactin impair the menstrual cycle in females and reduce the chances of conception (21). Peripheral cortisol levels can reflect adrenal function, which indirectly interferes with sex hormone production in infertile patients (17). In studies of IVF treatments, long-term cortisol levels measured from hair negatively predict pregnancy, and the accumulation of salivary cortisol accounts for 26.7% of the variance of clinical pregnancy outcomes (22). Lower serum and follicular cortisol levels on the day of oocyte retrieval were found to be significantly associated with successful IVF treatment (23). Although the mechanism remained unclear, cortisol is likely an important neurohormone in the complex relationship between psychosocial stress and IVF outcome (24).

Studies of physiological biomarkers for anxiety, however, revealed heterogeneous conclusions about cortisol dysregulations (14, 25). Women with anxiety demonstrated a higher level of 24-hour urinary free cortisol (26), but hair cortisol concentration representing the long-term secretion was not related to self-reported anxiety (27). The non-invasive collection of hair or urine samples is convenient for the patients, but the operation protocol involved complicated steps and instruments, which are rarely available in clinical settings (28, 29). In the contrast, blood sample collection is invasive, but already widely used for sex hormone level determination of infertile patients in hospitals (30). Elevation of morning serum cortisol level has been reported in pregnant women with anxiety, as determined by an automated commercialized chemiluminescence analyzer (31). Thus, to better demonstrate the correlation between cortisol dysregulation and anxiety, the variation in sampling preparation and test operation should be minimized.

This study aimed to evaluate the potential of serum morning cortisol levels in facilitating the diagnosis of anxiety in infertile women and investigate the influence of physiological and psychological stress on the IVF treatment outcome. Since most studies focused on the cortisol level at different time points during treatment, we would like to investigate whether psychological and physiological stress occurred before the treatment and affect the outcome of multiple IVF cycles. A Self-Rating Anxiety Scale (SAS) with a well-established Chinese norm and a novel point-of-care test (POCT) for serum cortisol was adopted. The standardized sample collection and convenient operation of the test platform enabled the rapid onsite detection of serum cortisol, and provide insights for future clinical applications. With the retrieval of the pregnancy outcome after IVF treatment, the influence of anxiety and cortisol dysregulation was addressed. We hypothesized that dysregulation of cortisol might be prevalent and linked to anxiety or poor pregnancy outcome in infertile women.

## Materials and methods

### Study design

This prospective cross-sectional study was conducted in the Second Affiliated Hospital of Chongqing Medical University from December 2018 to March 2020. The study design and protocol were in accordance with the Declaration of Helsinki (1989) and approved by the institutional review board of the hospital. Two cohorts were recruited, including 120 infertile women from the reproductive medicine center (infertility group) and 112 healthy women from the medical center (control group). The sample size of the study was calculated with an online calculator for clinical studies developed by Wang and Ji (32).

After obtaining informed consent, basic information including age, blood glucose, blood pressure, and regular blood test result of patients were checked for inclusion or exclusion. The infertile patients in the reproductive medicine center were already diagnosed before they were asked to join the study, whereas the healthy patients in the medical center were visiting the hospital for regular annual examinations. Since all volunteers were planning to undergo serum sample collection for other assays, they acknowledged that the sample would be shared for the cortisol test to minimize the risk. The blood collection was conducted between 7:00 am and 9:00 am. The infertility group completed an additional background information collection form, a questionnaire for anxiety, and a blood test for antral follicle count (AFC), anti-Mullerian hormone (AMH), and follicle-stimulating hormone (FSH) (Roche Cobas E601., Germany).

At the reproductive medicine center, 120 patients confirmed with infertility (failed to become pregnant after 1 year of preparation) were recruited. The inclusion criteria were as follows (1): 20 - 45 years old (2), able to understand and complete the SAS test, and (3) able to visit the reproductive medicine center before 7:30 am and finish blood collection on time (4), no hypertension or hyperglycemia, and (5) no visible abnormality in the blood sample. The exclusion criteria were as follows (1): under hormone treatment such as dexamethasone in the past 3 months (2); having endocrine diseases such as hypothyroidism, hyperprolactinemia, adrenal cortical hyperplasia, and diabetes (3), a history of psychological disorder (4), history of smoking or alcohol abuse. Patients in the control group have no known medical history of infertility treatments. The same inclusion criteria were adopted for these healthy individuals, except that they were only asked for the detection of morning serum cortisol. For the infertile women, those with severe negative life events within 1 year and known gynecological diseases such as uterine malformation and severe endometriosis were also excluded.

After completing all procedures, the date was matched and checked. Women who lacked background information or failed to complete any tests were excluded (n = 10), resulting in a final number of 110 (91.7%) and 112 (100%) for infertility and control groups, respectively. Among the 110 infertile patients, 1 didn’t undergo IVF treatment in our reproductive medicine center for personal reasons. A GnRH-antagonist protocol for IVF treatment was applied for all patients during the first cycle, which started within 1 month of the stress evaluation. More IVF cycles were conducted if the first one failed. The treatment continued until successful clinical pregnancy or the patient decided to give up. Finally, the number of IVF cycles performed and treatment outcomes were retrieved for the 109 participants.

### Measurement of cortisol

The collection of serum samples was conducted before breakfast with a standard extraction protocol and equipment. After arrival and resting for > 30 min in the morning, 5 mL of peripheral blood was taken between 7:00 am and 9:00 am using a standard coagulation blood collection tube. The sample was centrifuged at 3500 rpm for 6 min, and the serum was isolated for testing and storage. Serum cortisol was either measured immediately after separation or stored at 4°C before being tested within 8 h. The samples were kept at −20°C for long-term storage.

A quantum-dot immunochromatography assay for cortisol was used in this study (Jiangsu NepQD Biotech Ltd.). The quantitative result of serum cortisol concentration was measured by a portable immunofluorescence analyzer designed for this POCT (NepQD-Infinity-V1, Jiangsu NepQD Biotech Ltd., China). To perform the assay, 20 μL of serum sample was mixed with the diluent, and 60 μL of diluted sample was added to the sample loading well of the test card. After 10-min incubation, the test card was inserted into the analyzer for fluorescent signal reading. The detection range of this platform is 1.00–60.00 μg/dL, with an intra- and inter-assay coefficient of variation < 15%. The performance correlation between this POCT platform and the chemiluminescent assay is > 0.975.

### Measurement of SAS

A Chinese version of the widely employed SAS by Zung was used for the psychological evaluation of infertile patients (33). Basic instructions were provided by trained researchers using the same wording, and then the patients were asked to complete the 20-item questionnaire independently. The raw score was multiplied by 1.25 according to the Chinese norm, with the cutoff being 50. Scores of 50–59, 60–69, and above 70 indicate mild, medium, and severe levels of anxiety, respectively.

### IVF treatment

Patients involved in this study underwent GnRH-antagonist protocol for the first cycle of IVF treatment. Embryos transfers were performed on day 3 (2 cleavage stage embryos) or day 5 (1 blastocyst). Most patients finished the first IVF cycle within 3 months. Those who were not pregnant continued with other treatment protocols. The IVF treatment continued until successful clinical pregnancy or the patient decided to give up. In the end, the number of IVF cycles performed and treatment outcomes were retrieved for each individual.

### Statistical analysis

The data of 110 patients from the reproductive medicine center (infertility group) and 112 patients from the medical center (control group) were used for statistical analysis using SPSS Statistics 23.0 (IBM Corporation, Armonk, NY, USA). The distributions of age, serum cortisol level, BMI, SAS score, AFC, AMH, and FSH concentrations were examined using the Shapiro–Wilk test. Since the data were nonparametric, the differences in age and cortisol concentration between infertility and control groups were verified using the Mann–Whitney U test. The anxiety status of women with primary or secondary infertility was also compared with this method.

To investigate the level of cortisol across different ages, the participants were divided into different age groups (≤25, 26–28, 29–31, 32–34, and ≥35), and the difference was verified using the two-tailed Mann–Whitney U test. The correlation between variables was evaluated using the non-parametric hypothesis test Kendall test, with the significance level set at 0.05 and 0.01. Further comparison between age, cortisol level, monthly income, year of infertility, level of education, and BMI was performed between four anxiety groups (no, mild, medium, and severe) among the infertility participants using the Kruskal–Wallis test, which is a non-parametric test for median comparison between multiple groups.

The onset of anxiety among infertile patients was assessed based on the SAS questionnaire. Binary logistic regression was conducted to illustrate the contributing factors of anxiety. “Anxiety” and “no anxiety” was set as the dichotomous dependent variables, and age, cortisol level, year of infertility, BMI, monthly income, and education level were set as covariates. Monthly income and education level were categorical covariates, and others were numerical variants. The Forward: Likelihood Ratio model was selected for the construction of the regression equation.

To investigate the potential role of morning serum cortisol concentration in the evaluation of anxiety in the target population, we constructed the **receiver operating characteristic (**ROC) curve and determined the Youden index and cutoff value for cortisol. The sensitivity, specificity, positive predictive value (PPV), and negative predictive value (NPV) for anxiety were calculated based on the cutoff concentration. A Pearson’s Chi-square analysis was used to evaluate the difference in clinical pregnancy outcomes regarding the physiological and psychological approach for anxiety assessment, and the average number of IVF cycles conducted for each subgroup was calculated. The influence of infertility types and factors on pregnancy outcomes were also compared with the same method.

## Results

### Demographic characterization and cortisol levels

The basic information of individuals in the infertility group (N = 110) and control group (N = 112) is summarized in **Table 1**. The age of the infertility group ranged from 23 to 44 years with an average of 30.67 years, whereas that of the control group ranged from 21 to 44 years with an average of 31.37 years. In the infertility group, 20 (18.2%) patients were at the advanced maternal age as defined by the Chinese standard (≥35), and 15 (13.6%) were overweight according to local standards (BMI > 24.0) (34, 35). Patients at advanced maternal age generally have a decreased likelihood of natural pregnancy and a higher incidence of pregnancy complications (35). The year of infertility ranged from 1 to 8 years. Most of the patients (58.2%) that joined this study had a history of infertility of 2-4 years.

**Table 1:** Demographic characteristics of participants.

Parameters	Mean (SD)	Levels	Counts
Age			
Control group	31.38 ± 5.62^*^	≤25	17 (15.2%)
		26–34	63 (56.2%)
		Above 35	32 (28.6%)
Infertility group	30.67 ± 4.48^*^	≤25	10 (9.1%)
		26–34	80 (72.7%)
		Above 35	20 (18.2%)
Cortisol (μg/dL)			
Control group	14.38 ± 4.84		
Infertility group	18.39 ± 7.79^*^		
AFC	12.03 ± 5.26^*^		
AMH (ng/ml)	3.65 ± 2.51^*^		
FSH (mIU/mL)	7.81 ± 2.18		
SAS Score	38.45 ± 22.32^*^	0-49	64 (58.1%)
		50-59	17 (15.5%)
		60-69	19 (17.3%)
		Above 70	10 (9.1%)
BMI	21.51 ± 2.04^*^	<18.5	6 (5.5%)
		18.5–24.0	89 (80.9%)
		>24.0	15 (13.6%)
Years of infertility	3.74 ± 2.12^*^	1 Y	13 (11.8%)
		2–4 Y	64 (58.2%)
		Above 5 Y	33 (30.0%)
Monthly income (CNY)		Below 2000	17 (15.4%)
		2000–5000	51(46.4%)
		Above 5000	42 (38.2%)
Education level		Middle school/below	22 (20.0%)
		High school	41 (37.3%)
		Bachelor/above	47 (42.7%)

*Test for normal distribution, p < 0.05 (two-tailed).

As shown in **Table 1**, the serum cortisol level of the control group followed a normal distribution, but that of the infertility group did not. A comparison of the two groups revealed a significant difference in stress regulatory hormone concentration (p = 0.000), but not in age (p = 0.337). The level of morning cortisol was significantly higher in the infertility group (18.39 μg/dL), with a larger standard deviation ( ± 7.79 μg/dL).

### Correlation of variables

**Figure 1:**
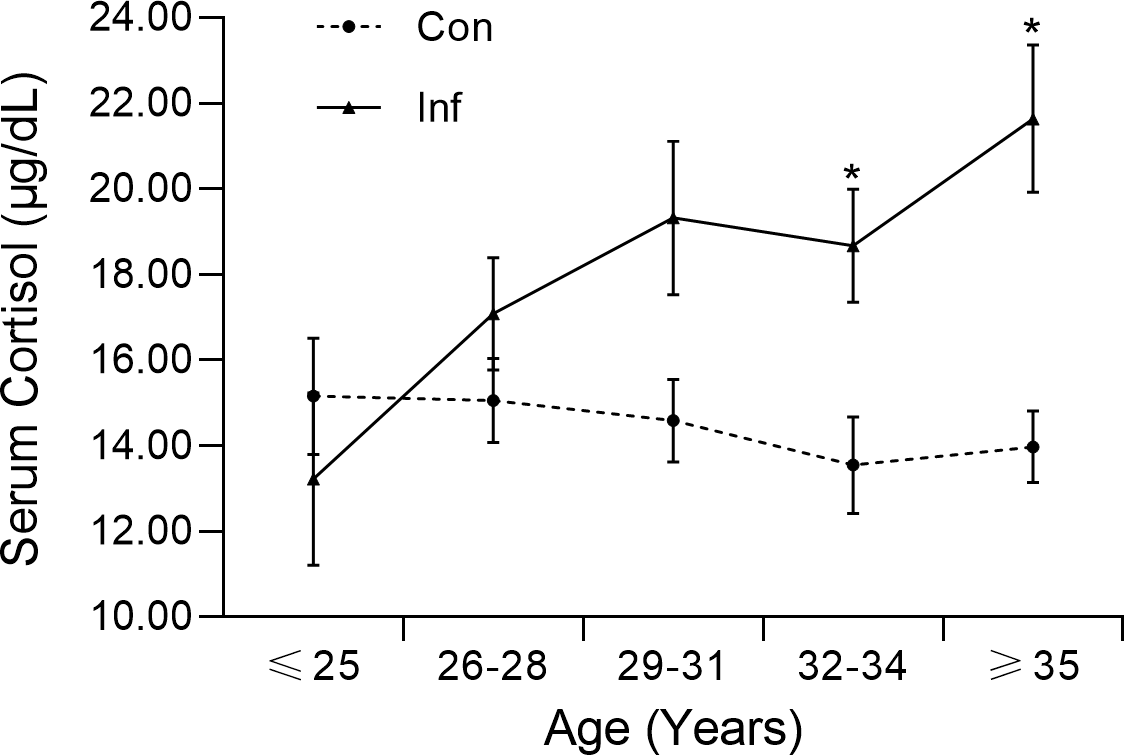
Morning serum cortisol level of infertile patients (Inf) and controls (Con) in different age groups. The error bar represents the standard error mean.

A positive correlation between cortisol level and age was found in the infertility group (Kendell’s tau = 0.201, p = 0.003), but not in the control group (Kendell’s tau = −0.064, p = 0.329). To further investigate the association between cortisol level and age, participants were divided into subgroups. The concentration gap between the two cohorts increased as their age increased, and a significant difference was observed for the 32-34 (Z = −2.725, p = 0.006) and >35 (Z = −3.630, p = 0.000) subgroups (**Figure 1**). The morning cortisol concentration was relatively stable across the age of interest (20–45) for the control patients.

Pairwise correlation analysis was conducted between different factors of the infertile patients (**Table 2**). The SAS score was strongly correlated to morning serum cortisol level, monthly income, and BMI (all p < 0.01). Cortisol concentration was positively related to age, SAS score, BMI (all p < 0.01), and year of infertility (p < 0.05), but was negatively related to monthly income (p < 0.01). Many demographic characters were correlated with each other, including age, year of infertility, BMI, and education level (all p < 0.01). Higher monthly income was linked to higher education levels but lower BMI (p < 0.01), which might be a reflection of healthier dietary habits. Interestingly, education level was negatively correlated to BMI and year of infertility (p < 0.01) in the infertility group. The number of cycles for IVF treatment was strongly related to age and year of infertility (p < 0.01), but negatively correlated to the pregnancy outcome (p < 0.05), which is because the treatment will continue until the patient gets pregnant or give up.

**Table 2:** Correlations between demographic and clinical variables of infertility group.

Correlation	SAS score	Cortisol level	Age	Monthly income	Year of infertility	BMI	Education level	IVF cycle
Cortisol level	0.624^**^							
Age	0.124	0.201^**^						
Monthly income	−0.232^**^	−0.248^**^	−0.150					
Year of Infertility	0.070	0.156^*^	0.495^**^	−0.153				
BMI	0.233^**^	0.299^**^	0.215^**^	−0.283^**^	0.152^*^			
Education level	−0.024	−0.075	−0.275^**^	0.303^**^	−0.240^**^	−0.258^**^		
IVF cycle	0.071	0.050	0.356^**^	-0.117	0.250^**^	0.123	−0.236^**^	
Pregnancy	-0.047	-0.087	-0.041	-0.033	-0.040	0.060	0.000	−0.212^*^

*p < 0.05 (two-tailed); **p < 0.01 (two-tailed).

### Prediction of anxiety with cortisol levels

As determined by the SAS score, 58.1% (n = 64) of infertile patients had no anxiety, whereas. Comparison between the anxiety (n = 46) and not anxiety (n = 64) groups of the infertility patients revealed that all numerical data (age, cortisol, years of infertility, BMI) of these two groups were in a skewed distribution. Further investigation of patients with different levels of anxiety (mild 15.5%, n = 17; medium 17.3%, n = 19; severe 9.1%, n = 10) demonstrated the pairwise difference in cortisol level, monthly income, and BMI (**Table 3**). The level of serum cortisol in patients with no anxiety was significantly different from that in patients with anxiety; meanwhile, patients with mild, medium, and severe anxiety had similar serum cortisol levels. The severe group had a significantly lower monthly income than other groups, implying that poverty might be a factor that exacerbates the disease. As the average BMI increased with the severity of anxiety, patients with no anxiety had a significantly lower BMI compared with the severe groups. This finding was generally in agreement with the percentage of overweight patients with no, mild, medium, and severe anxiety (4.7%, 17.6%, 21.1%, and 50.0%).

**Table 3:** Assessment of parameters among infertile patients with different levels of anxiety.

Pair of groups	Cortisol level	Age	Monthly income	Year of infertility	BMI	Education level
All groups	0.000^*^	0.071	0.007^*^	0.315	0.001^*^	0.292
No-mild	0.000^*^		1.000		0.073	
No-medium	0.000^*^		1.000		0.071	
No-severe	0.000^*^		0.003^*^		0.007^*^	
Mild-medium	1.000		1.000		1.000	
Mild-severe	1.000		0.112		1.000	
Medium-severe	1.000		0.060		1.000	

*Kruskal–Wallis test p < 0.05 (two-tailed); no, mild, medium, and severe represent the group of patients whose SAS score ranges from <50, 50–59, 60–69, and >70, n = 110.

To identify the factors that contributed to anxiety in the infertility group, we first conducted a binary logistic regression using the Forward: Likelihood Ratio model. Among all input factors, only cortisol level was significantly related to anxiety (*χ* ^2 =^ 93.307, p < 0.000). The regression analysis gave a sensitivity, specificity, and accuracy of 93.8%, 95.7%, and 94.5% respectively. This finding was in agreement with the significant difference (Z = −3.666, p = 0.000) in cortisol concentration between infertility and control groups (**Table 1**; **Figure 1**). In addition, the pairwise correlation test and binary logistic regression indicated a significant correlation between SAS score and morning serum cortisol concentration.

**Figure 2:**
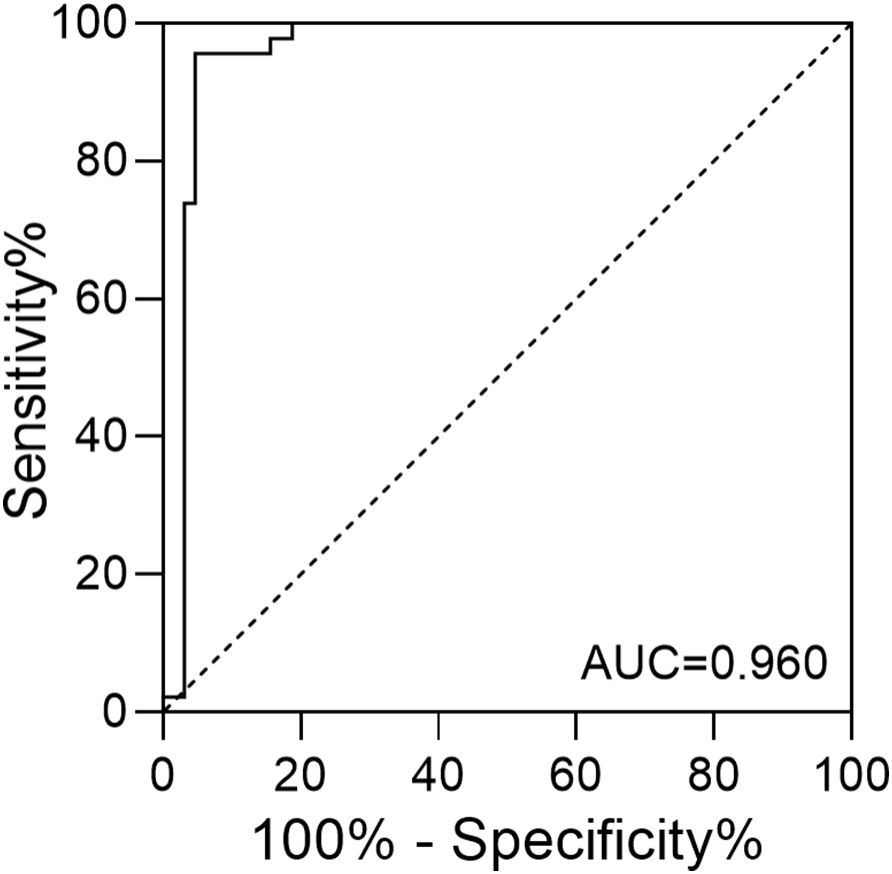
The ROC curve of serum cortisol concentration in predicting anxiety among infertile patients.

The prediction of anxiety with morning serum cortisol levels of the infertile women was achieved through the construction of the ROC curve. As shown in **Figure 2**, the area under the curve (AUC) was calculated to be 0.960, with a 95% confidence interval (CI) between 0.917 and 1.000. When the cutoff value for serum cortisol concentration was set to 22.25 μg/dL, the maximum value of the Youden index (0.910) was reached. Based on the threshold calculated, the performance of the cortisol test was summarized (**Table 4**). A PPV of 93.6%, NPV of 96.8%, sensitivity of 95.6%, and specificity of 95.3% were obtained. The overall diagnostic accuracy for the morning serum cortisol test to the SAS score was 95.4% for infertile patients.

**Table 4:** Performance of the serum cortisol level test in anxiety assessment.

		SAS score			
		Anxiety	No anxiety		
Serum cortisol	Anxiety	44	3	PPV	Sensitivity
		40.00%	2.73%	93.62%	95.65%
	Not anxiety	2	61	NPV	Specificity
		1.82%	55.45%	96.83%	95.31%
Total		46	64		

SAS cutoff value >50; Serum cortisol cutoff value > 22.25 μg/dL.

### IVF Treatment outcomes

Among the 110 infertile patients involved in the anxiety assessment, 109 underwent IVF treatment in our reproductive medicine center and 63.3% became clinically pregnant. Miscarriage was documented for one patient with primary infertility and two with secondary infertility. After 1 IVF cycle, 44.9% of the participants conceived, and 19.3% failed even with 2 cycles or more. As shown in **Table 5**, no significant difference regarding the final treatment outcome was found between patients with primary or secondary infertility, or between patients with various infertility factors. The pregnancy rate was similar between women with anxiety (58.7% and 57.4%) and without anxiety (66.7% and 67.7%), regardless of the method used for disease evaluation. When the cortisol levels and SAS scores of women who conceived during the first treatment cycle were compared, no significant differences were found. It should be noticed that after the first IVF cycle, the IVF treatment procedure varied between individuals to achieve the best outcome. Although a decrease in pregnancy rate (8.0%-10.3%) was observed for patients with high perceived anxiety (SAS > 50) or level of serum cortisol (concentration > 22.25 μg/dL), the impact of anxiety to multi-cycle IVF treatment was inconclusive.

**Table 5:** Comparison of the treatment outcome between patients with different types of infertility and infertility factors, as well as patients evaluated with SAS or morning serum cortisol.

	Group	Pregnant	Not pregnant	Total	IVF cycle	*χ2*	p-value
Type of infertility	Secondary	43 (61.43)	27 (38.57%)	70 (64.22%)	1.90	*0.296*	0.587
	Primary	26 (66.66%)	13 (33.33%)	39 (35.78%)	1.28		
Infertility factors	Husband	12 (57.14%)	9 (42.86%)	21 (19.27%)	1.33	*1.303*	0.728
	Wife	29 (60.42%)	19 (39.58%)	48 (44.04%)	1.58		
	Both	26 (70.27%)	11 (29.73%)	37 (33.94%)	2.03		
	Unknown	2 (66.66%)	1 (33.33%)	3 (2.75%)	1.33		
SAS	High SAS	27 (58.7%)	19 (41.3%)	46 (42.2%)	1.76	*0.727*	0.394
	Low SAS	42 (66.7%)	21 (33.3%)	63 (57.8%)	1.62		
Cortisol	High cortisol	27 (57.4%)	20 (42.6%)	47 (43.1%)	1.77	*1.220*	0.269
	Low cortisol	42 (67.7%)	20 (32.3%)	62 (56.9%)	1.61		

IVF cycle represents the average IVF cycle performed for the group of patients; SAS cutoff value > 50; Cortisol cutoff value > 22.25 μg/dL.

When comparing the number of IVF cycles performed, a significant difference was found between patients with primary and secondary infertility (1.28 VS 1.90). We also identified that women with secondary infertility were elder, and their SAS score, morning cortisol level, and BMI were significantly higher (p < 0.05) compared with those of primary infertility. However, if the anxiety statues were used as the independent variables, the average number of IVF cycles conducted for the anxiety groups was similar to that of the not anxiety ones (1.76-1.77 VS 1.61-1.62).

## Discussion

Demographic analysis between infertility and control groups revealed a similar age range and a skewed distribution. The morning serum cortisol demonstrated a normal distribution in that of the control group, which was similar to the distribution in the general population. Compare with hair cortisol which is also frequently evaluated in anxiety studies, serum sample requires fewer sample preparation steps and could be tested with commercialized platforms. More importantly, reference ranges have been proposed for serum/plasma cortisol levels of healthy individuals. As reported by the Roche Diagnostic GmbH (Roche Cobas platform, Elecsys Cortisol II), the 5^th^-95^th^ percental of the morning (6-10 am) serum cortisol level in healthy adults ranges from 6.02-18.4 μg/dL, and the reference range reported by Beckman Coulter cortisol test is 6.7-22.6 μg/dL. The test results of the control patients in our study generally followed a similar distribution within the same reference range. However, the POCT method enabled the onsite evaluation of serum cortisol with a share of the serum sample, and the result can be obtained within 15 min. A significant increase in morning cortisol levels was identified among infertile participants, which might be due to higher stress (i.e., anxiety) or other factors related to endocrine dysregulation.

Despite the similarity in age, the infertile patients demonstrated a higher average serum cortisol concentration and an increased cortisol level with age, especially in those over 32 years old (**Figure 1**). Previous studies have also documented higher cortisol levels in women with anxiety or patients with depression (31, 36). The gap in cortisol levels between the same age groups was possibly due to physiological or psychological differences between the two populations, as women might feel more stressed about their infertility issues in their 30s.

The correlation analysis for the primary variables of infertile patients revealed complicated relationships between variables (**Table 2**). Our result demonstrated a significant correlation between the SAS score and morning serum cortisol level, which has also been reported in pregnant women with anxiety (31). These findings implied that women with a stress-induced mood disorder were likely suffered from dysregulation of the stress-regulatory hormone as well.

The negative correlation between monthly income and SAS score was in agreement with several studies involving the socioeconomic status of patients with anxiety and depression (6, 10). A previous investigation conducted by Xu et al. in China revealed that higher education in infertile women could be a protective factor against anxiety (7). Although we did not observe a strong correlation between SAS score and education level, the significant correlation between education level and monthly income supported the same finding indirectly.

Based on the SAS score, the prevalence of anxiety in the infertility group was 41.9%, which is within the range of that of low- and middle-income countries (95% CI: 31.86%–78.62%) reported by Kiani et al. (4). The monthly income represents a typical medium income level in China, but the percentages of overweight patients in the mild, medium, and severe anxiety groups were much higher than that in the no-anxiety group, which was not related to the age difference (**Table 3**). The percentage of overweight women in the severe anxiety group was much higher than that of local women of childbearing age (37). Further investigation in a larger sample size is needed to identify the mechanism.

Due to the complicated physiological conditions of infertile patients, we could not determine whether the significant correlation between anxiety and BMI was caused by any minor malfunction of the endocrine system such as cortisol hypersecretion (p < 0.01) or the oily diet popular in the Chongqing region. Infertile anxiety women are generally prone to chronic pressure and are more likely to have a worse diet and unhealthy lifestyles, which exacerbate the issue. Similar to our result, a positive correlation between BMI and morning cortisol level has also been reported in a previous study (38). We postulate that hypercortisolemia could potentially contribute to increasing adiposity in the setting of caloric excess, as cortisol also regulates energy metabolism. Compared with serum cortisol level, which showed an increasing trend in the infertility group (**Figure 1**), the SAS score was independent of age. These findings suggest that the development of anxiety disorder in our patients might be attributed to both physiological and socioeconomic factors.

Our analysis also identified a positive connection between the year of infertility, age, and BMI. These findings were in agreement with the idea that being overweight (BMI > 24.0) might cause many health issues such as infertility (39). Neither the SAS score nor the cortisol level was associated with the year of infertility, suggesting that stress or anxiety may not exacerbate with time. However, the onset of mood disorder and the impairment of reproductive function might be connected through complicated endocrine issues.

A previous study involving both sides of infertility couples indicated that sex, education level, infertility duration, and treatment failure would all contribute significantly to anxiety (4). However, the binary logistic regression analysis in the present work suggested that only the morning serum cortisol level contributes significantly to anxiety (p = 0.000). The difference might be due to the lack of cortisol level as a parameter, the usage of different evaluation instruments (SAS vs Generalized Anxiety Disorder-7), the selection of the target population (diagnosed female vs couples), and the social or environmental difference. Our study suggested that morning serum cortisol levels yielded a high AUC for the ROC curve and predictive accuracy comparable to the binary logistic model (**Figure 2**; **Table 4**). The application of the POCT method suggested that morning serum cortisol level could serve as a potential biomarker to facilitate the screening of anxiety among infertile women.

The effect of stress on IVF outcomes has been investigated intensively in the field. It has been reported that both anxiety and concentrations of cortisol rose during IVF treatment (40), and lower blood cortisol at the time of oocyte retrieval is associated with successful treatment (24). Since our anxiety evaluation was conducted before any sex hormone administration and IVF treatment, it should be considered as a baseline for the psychological and physiological condition of patients. At this stage, the physiological condition of patients was not affected by external hormones, and they were not influenced by the excitement or stress of IVF treatment.

To further investigate the potential influence of anxiety on multi-cycle IVF treatment, the pregnancy outcome for 109 patients was retrieved. We found that the number of IVF cycles conducted was positively correlated with age (p < 0.01) and year of infertility (p < 0.01), but negatively correlated with pregnancy outcome (**Table 2**). This suggested that as the patients grow older, the difficulty of treatment increases, even if multiple procedures were tried. As shown in **Table 5**, the types and factors for infertility did not show a strong impact on the outcomes of the multi-cycle IVF treatment. However, patients with secondary infertility underwent more IVF cycles (1.90 VS 1.28), probably because the average age of these women was elder (31.6 VS 29.2). The significantly severe anxiety status of the elder, secondary infertility women evaluated through SAS and cortisol level also supports the idea that psychological evaluation and support should become incorporated into the medical service provided by reproductive medical centers.

The pregnancy rate for the “anxiety groups” either determined through SAS or cortisol level was 8.0%-10.3% lower, but the result did not reach a significant level or affirmative conclusion. Fortunately, the pregnancy rate of the anxiety groups and no anxiety groups all lay within the success rate reported by our hospital in the past few years (53% to 64%). Compared with other reproductive medical centers in China (success rate 50% to 65%), the outcomes of the IVF treatments conducted in this study were also reasonable. The same result was confirmed for the pregnancy rate after the first IVF cycle, where the same GnRH-antagonist protocol was adopted. Previous studies involving only one treatment cycle revealed a strong correlation between lower morning cortisol levels on the transplant day and successful pregnancy (41). We speculate that the anxiety level might change during treatment, and the stress regulation on the operation day should have a stronger effect. In addition, variables such as the number of oocytes retrieved, mature oocytes, and the number of embryos transferred were not strictly controlled between the IVF cycles and individuals. Consequently, no affirmation could be done on the effect of anxiety on the outcome of multi-cycle IVF treatment. A slightly higher average number of IVF cycle (1.76-1.77 VS 1.61-1.62) was also observed for the anxiety patients, in agreement with the fact that more IVF cycle was required until the patient was conceived or finally gave up. Most patients who failed after the first IVF cycle were willing to try multiple times, which might lead to a higher success rate. As we observed in this study, an alternative IVF protocol might be effective and result in clinical pregnancy. It is also possible that the medical operations of the women during IVF, especially the injection of sex hormones, had overcome the influence of anxiety and cortisol dysregulation to a certain degree (42). The expectation of a positive outcome from the treatment (43), and the encouragement from doctors and nurses could also help to release the stress.

Generally, our study has several strengths such as the novel application of onset serum cortisol detection and the fast entrance to IVF treatment. The evaluation of anxiety before any IVF treatment enabled the quantitative comparison of morning cortisol concentration between the healthy control group and the infertile women, which was missing from previous studies (24, 41, 44). The analysis between these two groups of participants provided a better demonstration of morning serum cortisol levels as a reflection of physiological stress. The SAS questionnaire could be easily applied in the reproductive medical center and has a well-defined Chinese norm. Both the SAS and cortisol tests revealed the baseline psychological and physiological condition of the patients without the influence of hormone injection during treatment. However, the limitations of this study should also be mentioned. Cortisol as one of the most important stress and metabolism regulatory hormones might also relate to other stress-related mood disorders such as depression. Since the reason for infertility is often complicated, the causation between anxiety, cortisol dysregulation, and infertility is unclear. We observed a high prevalence of overweight participants with no hypertension or hyperglycemia in this study. Detailed information on the endocrine function and metabolism of these women remained unclear. Future studies with larger population sizes and better control IVF treatment protocols should be conducted to address these issues.

## Conclusion

The investigation of physiological and psychological stress revealed a high prevalence of anxiety and a significant increase in morning serum cortisol levels related to the SAS score of infertile women. Although the effect of anxiety on multi-cycle IVF treatment was inconclusive due to the variations in treatment protocols, we observed a slightly lower rate of clinical pregnancy for the anxiety patients, who also showed higher levels of morning serum cortisol. A study with a larger scale, identical infertility factors, or standardized IVF treatment between patients might be needed for better evaluation of the potential effect of anxiety.

Despite that anxiety before treatment could not be viewed as a direct drawback for infertility treatment, given the high prevalence of the disease and dysregulation of cortisol secretion, evaluation and intervention of the psychological and physiological issues of anxiety are preferred. Some relaxation methods such as music therapy could be recommended, and patients with severe symptoms can be referred to a psychologist (45, 46). With the convenience of sampling and the POCT method, morning serum cortisol measurement could be a feasible approach to promote the primary evaluation of anxiety and related endocrine dysregulations among infertile women. Such assessment before IVF treatment might be beneficial to the relief of stress, restoration of the endocrine regulatory system, and improve the well-being of patients.

## Trial registration

This trial was approved by the institutional review board of The Second Affiliated Hospital of Chongqing Medical University, identifier: No. 2018 (100).

## Data availability statement

The original contributions presented in the study are included in the article/supplementary materials. Further inquiries can be directed to the corresponding author.

## Ethics statement

This trial was approved by the institutional review board of The Second Affiliated Hospital of Chongqing Medical University, identifier: No. 2018 (100). The patients/participants provided their written informed consent to participate in this study.

## Author contributions

HZ and YC conceived and designed the study. QL prepared the cortisol test platform and wrote the first draft of the paper with YC. YW, BN, and TF performed the data retrieving, processing, and analysis. HC and XK helped with questionnaire collecting, data recording, and blood sample collection in the reproductive medicine center. HZ performed the cortisol measurement and supervised all data collected in the hospital. All authors contributed to the article and approved the submitted version.
